# A case of undifferentiated pleomorphic rectal sarcoma occurring after radiation exposure

**DOI:** 10.1007/s12328-024-02026-6

**Published:** 2024-08-09

**Authors:** Makoto Eizuka, Yosuke Toya, Shun Yamada, Tomofumi Oizumi, Shunichi Yanai, Norihiko Kudara, Naoki Yanagawa, Tamotsu Sugai, Takayuki Matsumoto

**Affiliations:** 1 Division of Gastroenterology and Hepatology, Department of Internal Medicine, School of Medicine, Iwate Medical University, Yahaba, Iwate Japan; 2 Department of Gastroenterology, Iwate Prefectural Ofunato Hospital, Ofunato, Iwate Japan; 3 Department of Molecular Diagnostic Pathology, School of Medicine, Iwate Medical University, Yahaba, Iwate Japan

**Keywords:** Undifferentiated pleomorphic sarcoma, Radiation proctitis, Colonoscopy

## Abstract

A 72 year-old man was referred to our hospital for a detailed examination of a recurrent rectal polyp. He had past histories of surgery and radiation therapy for prostate cancer at the age of 66 and endoscopic excision of a rectal polyp at the age of 70. Colonoscopy revealed a semi-pedunculated lesion surrounded by friable mucosa, which was positive under positron-emission tomography-computed tomography. Histopathological examination of the endoscopically excised polyp revealed proliferation of atypical cells, characterized by strong pleomorphic or spindle morphology, which was immunohistochemically compatible with undifferentiated pleomorphic sarcoma. We diagnosed this case as sarcoma presumably associated with radiation proctitis.

## Introduction

Radiation therapy (RT) has become an integral part of cancer treatment [1, 2]. While RT is effective for treating cancer, it can also cause adverse events [3, 4], including radiation-induced sarcoma (RIS) in the late stage after RT [5]. RIS can occur at any irradiated site, and it requires resection or chemotherapy. To date, however, few cases of RIS in the gastrointestinal tract have been reported [5, 6]. We herein report a case of undifferentiated pleomorphic rectal sarcoma presumably associated with prior RT.

## Case report

**Fig. 1:**
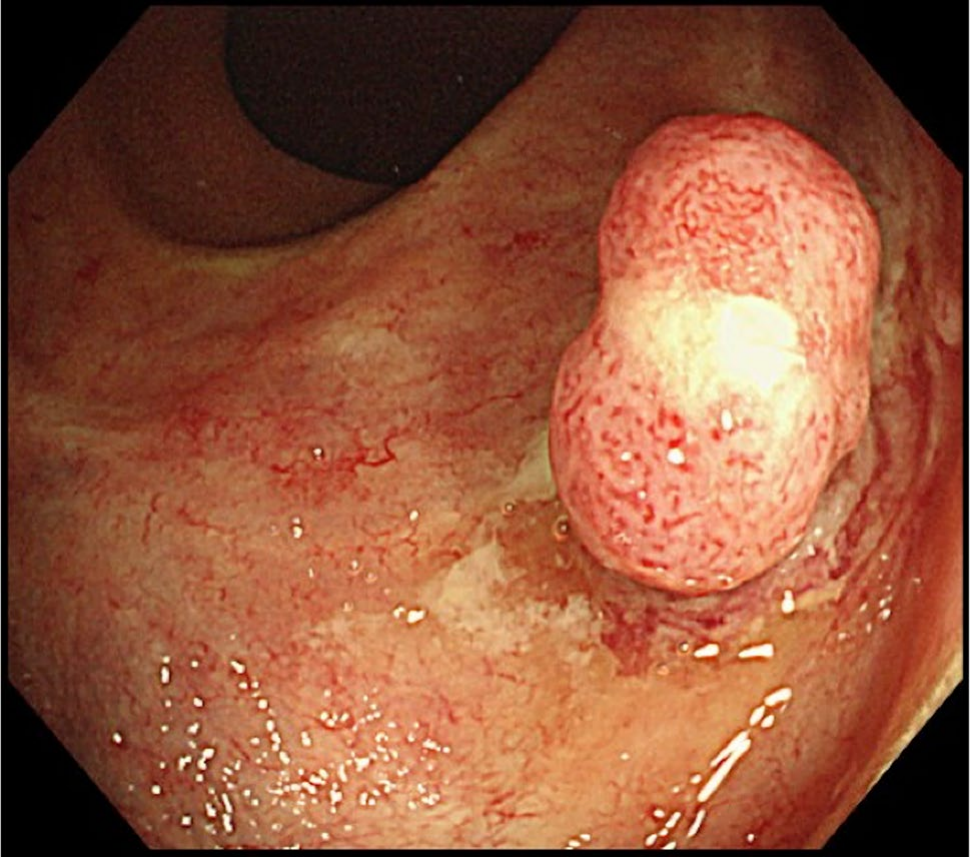
When the patient was 70 years of age, a colonoscopy revealed a 10 mm sized semi-pedunculated lesion in the rectum

**Fig. 2:**
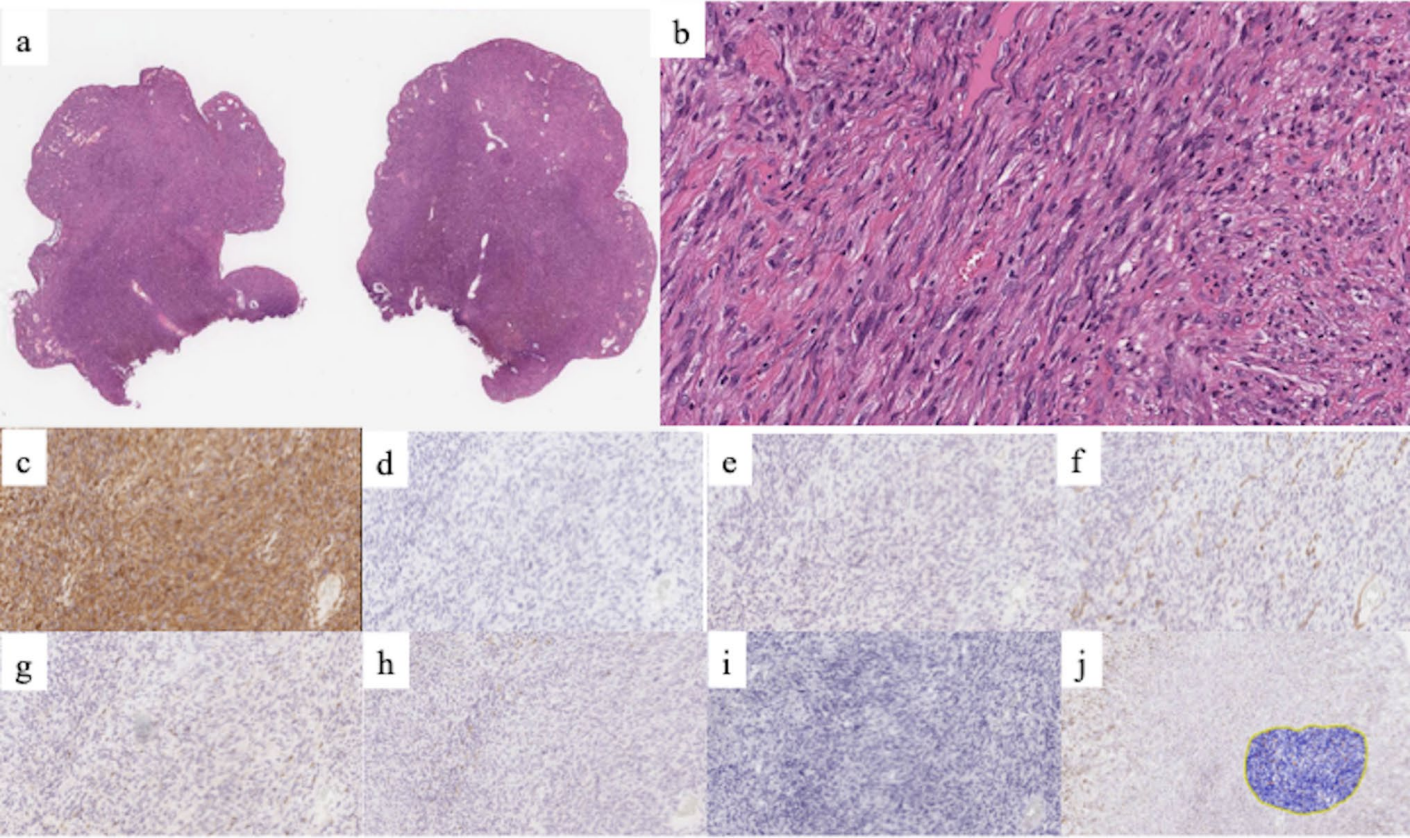
The previous resected specimen contained spindle-shaped atypical cells with nuclear pleomorphism proliferating in a fascicular pattern and the horizontal and vertical margins were undetermined (**a**, **b**). Immunohistochemistry showed that the specimen was positive for vimentin (**c**), while AE1/AE3 (**d**), desmin (**e**), SMA (**f**), KIT (**g**) S100 (**h**), and melan A (**i**) were negative. Ki-67 showed a scattered distribution, with an index of about 20% (**j**)

A 72 year-old man was referred to our hospital for recurrence of a rectal polyp that had been removed endoscopically. He had a history of surgery followed by RT (76 Gy, 38 fractions) for prostate cancer at the age of 66 years. He was subsequently diagnosed with radiation proctitis at the age of 68 years. At the age of 70 years, follow-up colonoscopy for radiation proctitis revealed a 10 mm, semi-pedunculated lesion in the rectum (Fig. 1), which was removed by endoscopic mucosal resection (EMR). The resected specimen contained spindle-shaped atypical cells with nuclear pleomorphism proliferating in a fascicular pattern, suggestive of a gastrointestinal stromal tumor (GIST). The horizontal and vertical margins remained undetermined for tumor invasion (Fig. 2a, b). Immunohistochemically, the atypical cells were positive for vimentin but negative for AE1/AE3, desmin, smooth muscle actin (SMA), KIT, S100, and melan A (Fig. 2c–i). The staining for Ki-67 showed a scattered distribution with an index of 20% (Fig. 2j).

**Fig. 3:**
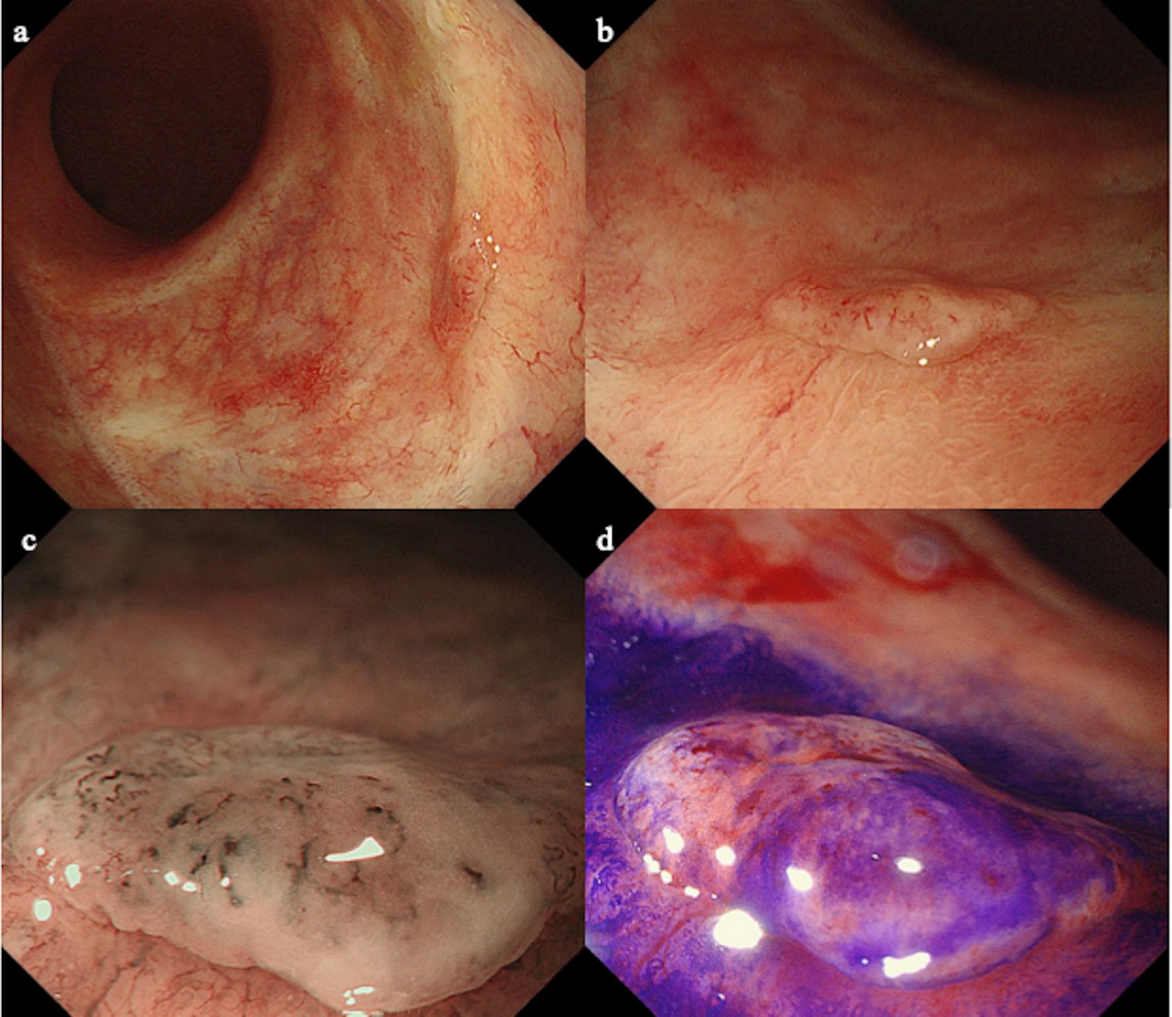
**a**, **b** When the patient was 72 years of age, colonoscopy identified a lesion that manifested as an 8 mm white, flat-elevated lesion with vascular dilation. **c** Magnifying endoscopy with narrow-band imaging showed dilated vessels. **d** Magnifying endoscopy with crystal violet staining showed that the superficial microstructure had disappeared

**Fig. 4:**
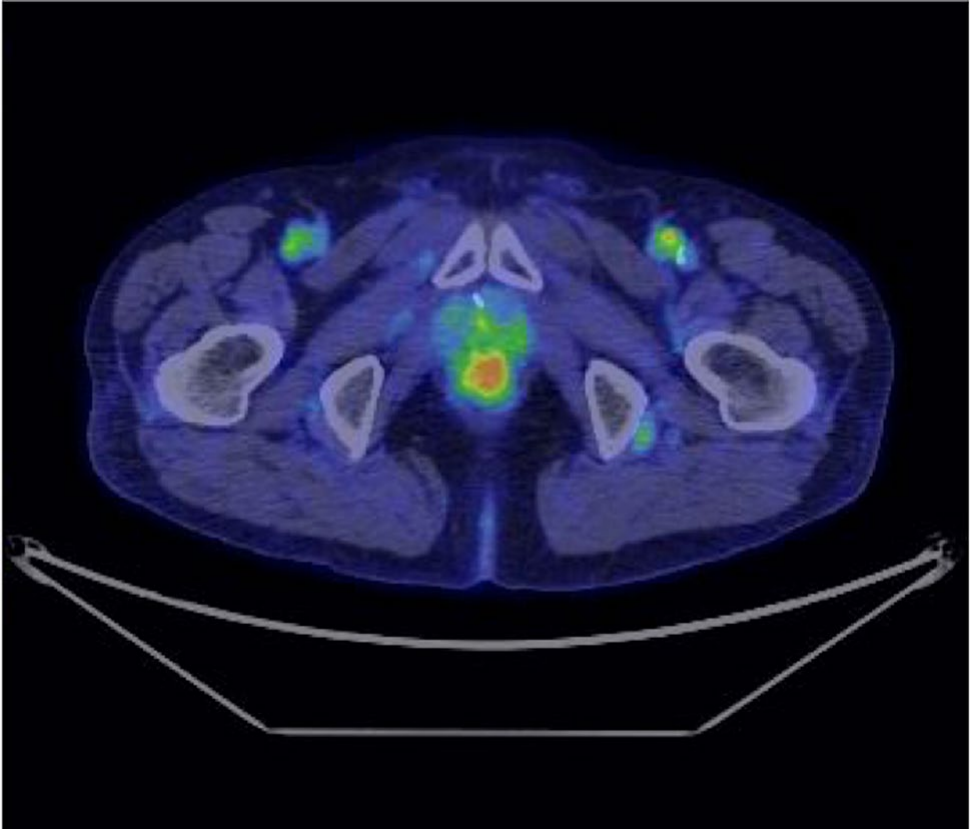
PET–CT detected FDG accumulation with a maximum standardized uptake value (SUV max) of 2.22 in the rectum

Follow-up colonoscopy 2 years after EMR revealed a flat, elevated lesion at the same site. Colonoscopy revealed the lesion to be an 8 mm, white, flat, elevated lesion with vascular dilation on the surface (Fig. 3a, b). Magnifying endoscopy showed vascular expansion without epithelial microstructure (Fig. 3c, d), suggesting a diagnosis of granulation tissue. On positron-emission tomography-computed tomography (PET-CT), an accumulation with a maximum standardized uptake value (SUV max) of 2.22 was found in the rectum (Fig. 4).

**Fig. 5:**
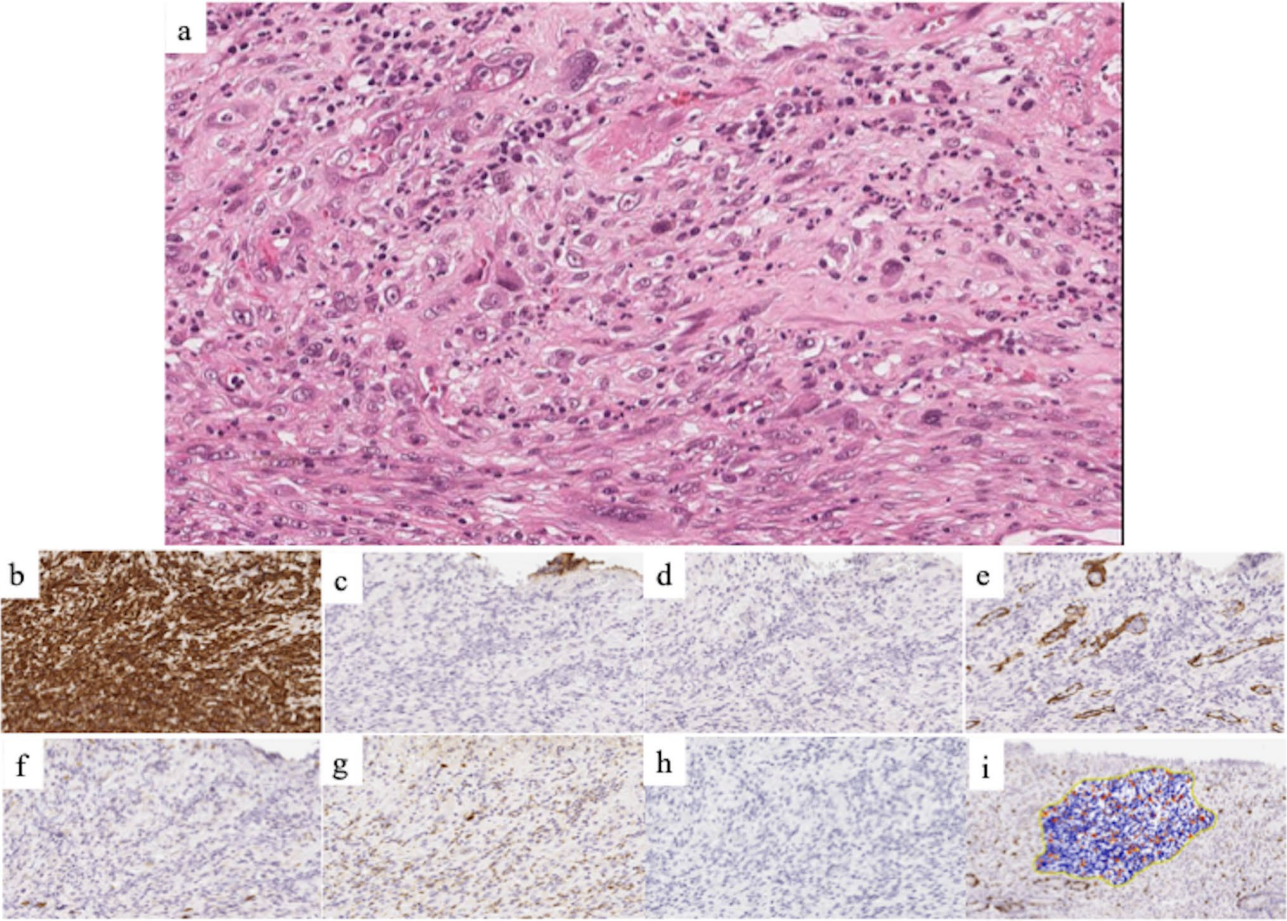
Histopathological examination revealed atypical cell proliferation with strong pleomorphism or spindle-shaped morphology, mainly in the proper mucosal layer to the submucosal layer (**a**). Immunohistochemistry showed that the specimen was positive for vimentin (**b**), while AE1/AE3 (**c**), desmin (**d**), SMA (**e**), KIT (**f**), S100 (**g**), and melan A (**h**) were negative. Ki-67 showed a scattered distribution, with an index of about 30% (**i**)

The endoscopic and PET–CT findings, as well as the location of the lesion at the site of radiation proctitis, suggested the lesion to be RIS. Because the lesion was small and had not metastasized to other organs, endoscopic submucosal dissection (ESD) was performed with the patient’s consent. Histopathological examination of the resected specimen revealed that the protruding lesion was composed of proliferating atypical cells of pleomorphic or spindle-shaped morphology, primarily within the proper mucosal layer and invading the submucosal layer (Fig. 5a). Immunohistochemically, the cells showed the identical patterns as seen in the previously resected specimen (Fig. 5b–h). Ki-67 showed a scattered distribution with an index of about 30% (Fig. 5i). Based on these findings, we diagnosed the lesion as high-grade, undifferentiated pleomorphic sarcoma (UPS). The horizontal and vertical margins of the resected specimen were free of tumor cells. In the surrounding non-neoplastic mucosa, crypts and goblet cells were preserved, and inflammatory cell infiltration was observed in the proper mucosal layer. Based on these findings, a diagnosis of RIS was established.

Six months later, the patient underwent repeated PET–CT and colonoscopy, which revealed no recurrence or metastasis. He has been under observation with annual PET–CT and colonoscopy.

## Discussion

In the past, RIS was reported to be a late adverse event after RT [3–8]. The diagnostic criteria for RIS are prior radiation exposure, subsequent development of sarcoma within the irradiated tissue, and histologic confirmation of sarcoma distinct from the original primary malignancy [7, 8]. Our patient had prior radiation exposure for prostate cancer and developed radiation-induced proctitis. Subsequently, an undifferentiated pleomorphic sarcoma was found at the site of rectal irradiation. The patient’s clinical course fulfilled the diagnostic criteria for RIS.

In our case, the resected specimen at the age of 70 years showed spindle-shaped atypical cells, which were suggestive of GIST. However, the immunohistochemical findings were not compatible with the diagnosis. In the second endoscopically resected specimen, the histological and immunohistochemical findings remained unchanged. Because the involvement of the resected margin in the first specimen remained obscure, we believe the second lesion to be a local recurrence.

In 2010, Gladdy et al. reported the clinicopathological features of 130 cases of RIS. They found that the median age at diagnosis of RIS was 59 years, with a range from 18 to 86 years, and the median interval from the start of RT to the diagnosis of RIS was 10 years, with a range from 1.3 to 74 years. In their cohort, 108 of 130 RISs (83%) were histologically compatible with high-grade sarcoma, and the predominant tumor site was the trunk (61.5%), as opposed to the extremities and the abdomen [9]. Furthermore, the most common histologic type of RIS was pleomorphic malignant fibrous histiocytoma (MFH) (*n* = 34 [26%]), followed by angiosarcoma (*n* = 27 [21%]), leiomyosarcoma (*n* = 16 [12%]), fibrosarcoma (*n* = 16 [12%]), malignant peripheral nerve sheath tumor (MPNST) (*n* = 11 [9%]) and liposarcoma (*n* = 4 [3%]) [9]. Since MFH does not show clear histiocytic differentiation, the nomenclature “MFH” was subsequently revised to UPS [10]. UPS is now widely used as a generic term for many unclassifiable malignant soft tissue tumors that lack specific genetic characteristics or differentiation trends that can be demonstrated by genetic or histological examination [10]. More recently, Kim E, et al. attempted whole genome sequencing in 11 cases of RISs and compared the result with that found in spontaneous sarcoma genomes. As a consequence, the investigators found that the nonhomologous end joining pathway for DNA damage repair may be associated with the tumorigenesis of RIS [11]. Other genetic features of RIS need to be elucidated further.

RIS occurs predominantly in the deep soft tissues of the trunk, whereas gastrointestinal involvement is extremely rare. In fact, our online search of PubMed during the period from 2004 to 2024 identified only four cases of rectal RIS (Table 1) [12–15]. Among those cases, the primary cancers requiring RT were of gynecological or urological origin (cervical cancer in three cases and prostate cancer in one case). The median time from RT to diagnosis of RIS was 10.5 years, with a range from 8 to 32 years. All cases of RIS were resected surgically, and the histological type was found to be leiomyosarcoma in three cases and angiosarcoma in one case. We removed our patient’s rectal lesion by ESD based on its extremely small size and presumably less invasive involvement in the rectal wall. To the best of our knowledge, this is the first report of gastrointestinal RIS treated by endoscopic resection.

**Table 1:** Published cases of radiation-induced sarcoma of the rectum

Reference	Age, years, gender	Primary disease	Time period from radiation exposure to onset of RIS, years	Treatment	Histologic type	Size, mm
Ahmad A, et al. [12]	74, male	Prostate cancer	8	Resection	Angiosarcoma	80
Jayakumar R, et al. [13]	58, female	Cervical cancer	13	Resection	Leiomyosarcoma	40
Garcia–Ortega DY, et al. [14]	58, female	Cervical cancer	8	Resection and chemotherapy	Leiomyosarcoma	70
Makhmudov DE, et al. [15]	62, female	Cervical cancer	32	Resection	Leiomyosarcoma	45
Present case	72, male	Prostate cancer	4	ESD	UPS	8

*ESD* endoscopic submucosal dissection, *UPS* undifferentiated pleomorphic sarcoma

Descriptions of the endoscopic features of gastrointestinal RISs are sparse. The initial endoscopic findings of the tumor in our case were a semi-pedunculated and reddish polyp with obvious vasculature on the surface. Hypervascularity was also found at the time of recurrence. These observations suggested that mesenchymal tumor, lymphoma, melanoma and condyloma acuminatum were the candidate differential diagnoses for rectal RIS. In this regard, a reddish protrusion occurring in rectal mucosa damaged by prior irradiation may be specific to the endoscopic diagnosis of RIS.

It should be noted that the overall prognosis of RIS remains to be established. Cha et al. reported that the 5 year survival rate of RIS was 41%, with a median survival time of 48 months [16]. They also reported that histologically high-grade tumor, patient age > 60 years, and positive resection margins were predictors of poor prognosis [16]. In our case, histological examination of the resected specimen revealed high tumor cellularity, which seemed to be high-grade UPS, but the resected margin was free of tumor cells.

In conclusion, we report a case of rectal RIS that was histologically compatible with UPS. RIS should be considered for polypoid lesions occurring in the gastrointestinal tract damaged by prior RT.
